# Identification of Risk Groups for and Factors Affecting Metabolic Syndrome in South Korean Single-Person Households Using Latent Class Analysis and Machine Learning Techniques: Secondary Analysis Study

**DOI:** 10.2196/42756

**Published:** 2023-09-12

**Authors:** Ji-Soo Lee, Soo-Kyoung Lee

**Affiliations:** 1 Department of Nursing Gimcheon University Gimcheon-si Republic of Korea; 2 Big Data Convergence and Open Sharing System Seoul National University Seoul Republic of Korea

**Keywords:** latent class analysis, machine learning, metabolic syndrome, risk factor, single-person households

## Abstract

**Background:**

The rapid increase of single-person households in South Korea is leading to an increase in the incidence of metabolic syndrome, which causes cardiovascular and cerebrovascular diseases, due to lifestyle changes. It is necessary to analyze the complex effects of metabolic syndrome risk factors in South Korean single-person households, which differ from one household to another, considering the diversity of single-person households.

**Objective:**

This study aimed to identify the factors affecting metabolic syndrome in single-person households using machine learning techniques and categorically characterize the risk factors through latent class analysis (LCA).

**Methods:**

This cross-sectional study included 10-year secondary data obtained from the National Health and Nutrition Examination Survey (2009-2018). We selected 1371 participants belonging to single-person households. Data were analyzed using SPSS (version 25.0; IBM Corp), Mplus (version 8.0; Muthen & Muthen), and Python (version 3.0; Plone & Python). We applied 4 machine learning algorithms (logistic regression, decision tree, random forest, and extreme gradient boost) to identify important factors and then applied LCA to categorize the risk groups of metabolic syndromes in single-person households.

**Results:**

Through LCA, participants were classified into 4 groups (group 1: intense physical activity in early adulthood, group 2: hypertension among middle-aged female respondents, group 3: smoking and drinking among middle-aged male respondents, and group 4: obesity and abdominal obesity among middle-aged respondents). In addition, age, BMI, obesity, subjective body shape recognition, alcohol consumption, smoking, binge drinking frequency, and job type were investigated as common factors that affect metabolic syndrome in single-person households through machine learning techniques. Group 4 was the most susceptible and at-risk group for metabolic syndrome (odds ratio 17.67, 95% CI 14.5-25.3; *P*<.001), and obesity and abdominal obesity were the most influential risk factors for metabolic syndrome.

**Conclusions:**

This study identified risk groups and factors affecting metabolic syndrome in single-person households through machine learning techniques and LCA. Through these findings, customized interventions for each generational risk factor for metabolic syndrome can be implemented, leading to the prevention of metabolic syndrome, which causes cardiovascular and cerebrovascular diseases. In conclusion, this study contributes to the prevention of metabolic syndrome in single-person households by providing new insights and priority groups for the development of customized interventions using classification.

## Introduction

### Background

Single-person households have rapidly increased from 9% in 1990 to 29.3% in 2018, accounting for one-third of all South Korean households [1], and are estimated to reach approximately 36.3% by 2045 [2]. This increasing trend is also evident worldwide, including the United States (26.7%), Australia (23.9%), and Japan (32.4%) [3,4].

The reasons for this rising trend include the large number of unmarried people and late marriages, resulting in changes in marital values, divorce, separation, high unemployment, and diverse and complex social factors in larger cities [5]. On the basis of individuals’ sociodemographic characteristics and lifestyle, single-person households are more susceptible to exposure to high-risk health behaviors, such as smoking and alcohol consumption, as well as experiences of depression and stress, than multiperson households [6-8].

Adult single-person households are known to show distinct differences from multiperson households in terms of demographic characteristics and living habits. For instance, it has been reported that single-person households are more likely than multiperson households to be more susceptible to health problems [9-11]. In addition, compared with multiperson households, single-person households are more exposed to high-risk health behaviors, such as smoking and drinking, and experience more depression and stress [12,13].

These sociodemographic characteristics and lifestyles indicate that single-person households have a higher prevalence of metabolic syndrome and chronic diseases, such as hypertension, diabetes, dyslipidemia, arthritis, asthma, myocardial infarction, and stroke [14-16].

Metabolic syndrome leads to cardiovascular disease and a risk of diabetes [6], involving at least 3 clinical characteristics, namely hypertension, hyperglycemia, and hypertriglyceridemia, and high levels of low-density lipoprotein, as well as to abdominal obesity [6,15]. It also increases the occurrence of myocardial infarction, stroke, and dementia [1,6,11,17]; therefore, it is important to decrease the incidence of metabolic syndrome to prevent chronic cardiac and cerebrovascular diseases and reduce the mortality rate [18,19].

It is also necessary to assess the morbidity associated with the disease and develop customized medications and guidelines to manage its risk factors [20]. Previous studies have demonstrated that risk factors include age, sex, obesity, smoking, a lack of physical activity, and education [4-7] Although single-person households include various characteristics, their influences on metabolic syndrome may differ from those of multiperson households and across age groups [1,3]. This necessitates a more holistic and systematic understanding of the metabolic syndrome risk factors in single-person households [1], as each risk factor may have a discriminatory or an interrelated effect on metabolic syndrome depending on individual characteristics [21].

Latent class analysis (LCA), a human-centered approach, checks the multidimensional characteristics of human behavior; it contrasts with a conventional variable-centered approach, which describes predictors’ relative influence on outcome variables [22-24]. In addition, identifying the patient type and characteristics is advantageous in predicting the disease, and a customized intervention program can be planned according to individual risk factor vulnerabilities and diagnosis [25-27]. Machine learning refers to a method of automatically extracting general rules or new knowledge by implementing learning ability, one of the unique intelligence functions of humans, through machines and analyzing the given data [28,29]. In this study, the factors affecting metabolic syndrome in South Korean single-person households were analyzed using logistic regression (LR), decision tree (DT), random forest (RF), and extreme gradient boost (XGBoost). LR, DT, and RF are the most commonly used machine learning techniques, and XGBoost is a machine learning technique that has recently emerged [27-29].

This study aimed to identify the factors affecting metabolic syndrome in single-person households using machine learning with large-scale health data from the National Health and Nutrition Examination Survey (NHANES) [30]. However, few studies have applied machine learning and LCA to identify the factors affecting metabolic syndrome in single-person households [23,24,30]. The contribution or significance of this study is not finding any exact answer but finding new variables or overlooked parts through basic research or translational research for clinical application. The core value of translational research lies in its effort to apply basic research to clinical practice with a high success rate at a low cost in a short period.

Hence, this study was designed to establish basic data to develop customized interventions by categorizing and characterizing metabolic syndrome risk factors in South Korean single-person households using machine learning techniques and LCA.

### Purpose of This Study

This study used data from the NHANES spanning 10 years (2009-2018), applied machine learning techniques to identify the factors that affect the occurrence of metabolic syndrome, and applied LCA to classify single-person households. The purpose of this study was to categorize risk groups and identify risk factors for metabolic syndrome in South Korean single-person households.

## Methods

### Research Design

**Figure 1:**
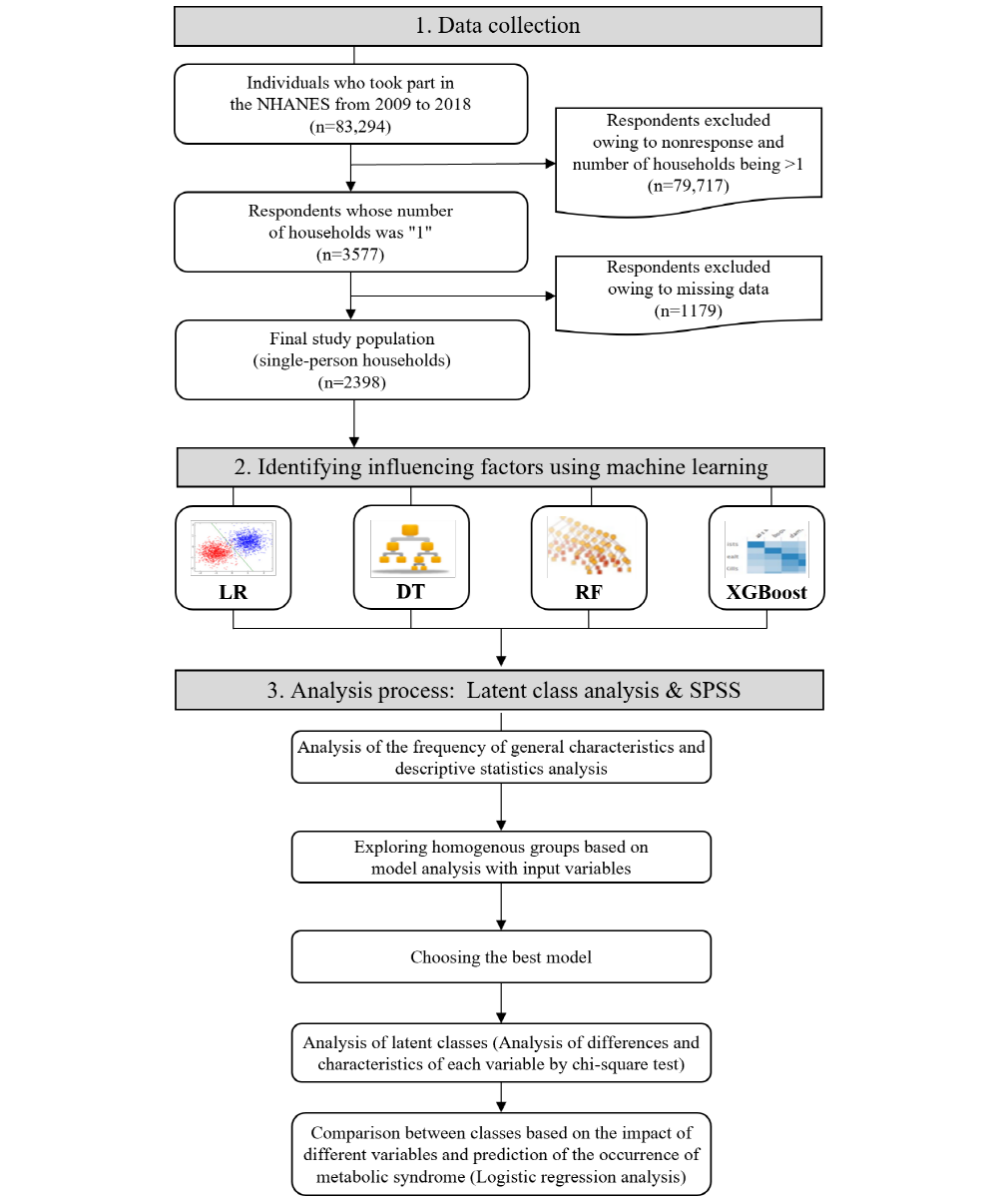
Overall flowchart of this study. DT: decision tree; LR: logistic regression; NHANES: National Health and Nutrition Examination Survey; RF: random forest; XGBoost: extreme gradient boost.

This study was a secondary data analysis that used machine learning techniques and LCA to categorize metabolic syndrome risk factors to identify the factors influencing the occurrence of metabolic syndrome in single-person households. The overall flowchart of the study is shown in Figure 1.

### Participants of the Study

This study used raw data from the 10-year NHANES (2009-2018) conducted by the Ministry of Health and Welfare and the Korea Centers for Disease Control and Prevention for a secondary data analysis. The South Korean NHANES generated data representative of the South Korean population using stratified colony sampling. The total number of respondents was 83,294, among whom there were 1376 (1.65%) single-person households, and 79,717 (95.71%) households with ≥2 persons. Of the 1376 single-person households, 1371 (99.64%) were finally selected as study participants, excluding 5 households because of missing data and older age.

### Data Set

We used the health questionnaire from the NHANES’s fourth (2009), fifth (2010-2012), sixth (2013-2015), and seventh terms (2016-2018).

#### General Characteristics

We selected participants with the following characteristics: sex (male or female), age (early adulthood, ie, 19-39 y of age, and middle adulthood, ie, 40-64 y of age), educational level (lower than high school to higher than an undergraduate [4-year] college degree), marital status (married or unmarried), income level, and economic activity status (active or inactive).

#### Health Behavior

We selected smoking behavior (smoking or nonsmoking); alcohol consumption (abstaining, <4 times/mo, or >2 times/wk); exercise, such as walking (<3 times/wk or >3 times/wk); subjective recognition of body type (very thin, slightly thin, normal, slightly obese, or very obese); subjective health status (very good, good, normal, bad, or very bad); and obesity status indicated by BMI (<18.5=underweight, 18.5-22.9=normal, 23-24.9=at risk, or ≥25=obese).

#### Eating Habits

We administered a questionnaire to determine how frequently respondents dined out (5 times/wk, 1-4 times/wk, and <3 times/mo) and their dietary lifestyle (“good” or “bad”).

#### Mental Health

We assessed respondents’ awareness of stress (recognition or nonrecognition) and diagnoses of depression (diagnosed or undiagnosed).

#### Use of Medical Institutions and Community Services

We classified participants based on health, cancer, and oral cavity using “yes” or “no” responses and included the type of health insurance (local, employment-related, or uninsured or self-paying medical care) and subscription to private medical insurance (registered or unregistered for private medical insurance).

#### Metabolic Syndrome

We determined the presence of metabolic syndrome based on the National Cholesterol Education Program–Adult Treatment Panel 3 diagnostic criteria [31] and whether respondents possessed ≥3 of the following 5 criteria: hypertension, hyperglycemia, hypertriglyceridemia, hypo–high-density lipoproteinemia, and abdominal obesity. Waist circumference, triglycerides, high-density lipoprotein cholesterol levels, final systolic and diastolic blood pressures (mean of the second and third measurements), and fasting blood glucose level were used to determine the existence of metabolic syndrome.

### Data Collection Method

We submitted our affiliation and purpose of using the data to the Korea Disease Control and Prevention Agency’s data portal and then used the data, which contained no personal information.

### Data Preprocessing

After sampling and merging the 10-year data from the NHANES, we conducted a data-cleansing process, and the distribution of variables was confirmed using the missing values function of the SPSS software (version 25.0; IBM Corp) to identify both ideal values and missing data [32].

In this study, data from a total of 83,294 individuals who participated in the 10-year (2009-2018) NHANES and application year survey were extracted. After extracting cases where the number of households (code name=*cfam*) was “1,” out of 83,294 households, we found 3577 (4.29%) single-person households from 2009 to 2018. Of these 3577 individuals from single-person households, 1371 (38.33%) were finally selected after excluding older adults (aged ≥65 y) and those with missing values.

After extracting 10 years of data from the NHANES, this study went through a lightweight process, and to check outliers and missing values in the data, the missing value program of SPSS was used to check the weight of the group. A total of 1182 cases were finalized, processed, and deleted to confirm the initial and intermediate defects applied in the overlapping files of 10 years of data from the NHANES. For the analysis, age, a continuous variable, was converted into a categorical variable, and a metabolic syndrome variable was newly created in the case of having at least 3 of hypertension, hyperglycemia, high-density lipoproteinemia, hypertriglyceridemia, and abdominal obesity. The case of having 3 or more of each currency was made a reimbursement syndrome. Metabolic syndrome was analyzed according to the National Cholesterol Education Program–Adult Treatment Panel 3 diagnostic criteria [31].

In this study, when the influencing factors of the syndrome were analyzed by applying LR, DT, RF, and XGBoost among machine learning methods, the total number of discussions of the 10-year data from the NHANES was 7450. From 2009 to 2018, there were 390 results of splitting the data using the 10-fold cross-validation method. Among them, 154 items that accumulated drainage, 5 diagnostic criteria for metabolic syndrome unrelated to measurements, and study participants were analyzed as factors influencing the occurrence of metabolic syndrome in single-person households based on the code name *MetS* (metabolic syndrome or not).

We applied LR, DT, RF, and XGBoost algorithms among machine learning techniques with a total of 7450 variables of the 10-year NHANES data to analyze the influencing factors of metabolic syndrome.

### Statistical Analysis

Data were analyzed using SPSS, Mplus (version 8.0; Muthen & Muthen), and Python (version 3.0; Plone & Python).

### Ethical Considerations

We performed data analysis after obtaining approval from Keimyung University’s ethics committee for an exemption from deliberation (institutional review board number 40525-202008-HR-043-01) because we used existing data or published documents instead of directly engaging with participants.

## Results

### Respondents’ General Characteristics

Of the 1371 respondents, 681 (49.67%) were male, 893 (65.13%) were middle-aged adults, and 807 (58.86%) had less than a high school education. Further, among the 1371 respondents, 990 (72.21%) had active economic activity, and 384 (28.01%) had low or intermediate income levels. Of the 1371 respondents, 705 (51.42%) were married, 932 (67.98%) were nonsmokers, and 749 (54.63%) consumed alcohol <4 times a month. Moreover, of the 1371 respondents, 938 (68.42%) walked >3 times a week, 518 (37.78%) considered themselves to have a normal body weight, 668 (48.72%) were subjectively healthy, and 541 (39.46%) recognized their subjective body type.

In addition, of the 1371 respondents, 602 (43.91%) and 778 (56.75%) respondents indicated that their father and mother had an elementary school education, respectively. Regarding mental health, 930 (67.83%) of the 1371 respondents were not aware of stress, and 1247 (90.96%) of the 1371 respondents were not diagnosed with depression (Table 1).

**Table 1:** General characteristics of the study participants (N=1371).

Variable		Participant, n (%)
**Sex**		
	Male	681 (49.67)
	Female	690 (50.33)
**Age (years)**		
	Early adulthood (19-39)	478 (34.87)
	Middle adulthood (40-64)	893 (65.13)
**Educational level**		
	≤High school	807 (58.86)
	≥College	564 (41.14)
**Economic activity status**		
	Active	990 (72.21)
	Inactive	381 (27.79)
**Income level**		
	Low	340 (24.8)
	Lower intermediate	384 (28.01)
	Upper intermediate	326 (23.78)
	Advanced	321 (23.41)
**Marital status**		
	Married	705 (51.42)
	Single	666 (48.58)
**Smoking**		
	Smoker	439 (32.02)
	Nonsmoker	932 (67.98)
**Frequency of drinking**		
	None	260 (18.96)
	<4 times/mo	749 (54.63)
	>2 times/wk	362 (26.4)
**Days of walking**		
	<3 times/wk	433 (31.58)
	>3 times/wk	938 (68.42)
**Obesity status**		
	Underweight	58 (4.23)
	Normal	518 (37.78)
	Overweight	339 (24.73)
	Obese	456 (33.26)
**Subjective health status**		
	Very healthy	59 (4.3)
	Healthy	285 (20.79)
	Normal	668 (48.72)
	Unhealthy	286 (20.86)
	Very unhealthy	73 (5.32)
**Subjective body shape recognition**		
	Very thin	41 (2.99)
	Little thin	162 (11.82)
	Normal	541 (39.46)
	Little obese	478 (34.87)
	Very obese	149 (10.87)
**Recognition status of stress**		
	No recognition	930 (67.83)
	Recognition	441 (32.17)
**Depression diagnosis by physician**		
	Negative	1247 (90.96)
	Positive	124 (9.04)
**Dietary condition**		
	Good	1267 (92.41)
	Poor	104 (7.59)
**Frequency of eating out**		
	2 times/d->5 times/wk	731 (53.32)
	1 time/wk-4 times/wk	398 (29.03)
	<3 times/mo	242 (17.65)
**Father’s educational level**		
	Elementary school graduate	602 (43.91)
	Middle school graduate	204 (14.88)
	High school graduate	335 (24.43)
	College graduate	230 (16.78)
**Mother’s educational level**		
	Elementary school graduate	778 (56.75)
	Middle school graduate	197 (14.37)
	High school graduate	290 (21.15)
	College graduate	106 (7.73)
**Health checkup status**		
	Yes	877 (63.97)
	No	494 (36.03)
**Cancer checkup status**		
	Yes	633 (46.17)
	No	738 (53.83)
**Oral examination**		
	Yes	898 (65.5)
	No	473 (34.5)
**Type of health insurance**		
	Regional health insurance	491 (35.81)
	Company health insurance	757 (55.22)
	Medical care	123 (8.97)
**Private health insurance**		
	Joined	1066 (77.75)
	Not joined	305 (22.25)

### Analysis of the Factors Influencing Metabolic Syndrome Using Machine Learning Techniques

We observed 390 common variables from 10 years of merged data (2009-2018) of the NHANES. Among them, 154 were excluded because they did not comply with the study, and 236 missing variables were analyzed to assess the factors affecting metabolic syndrome in single-person households.

Overall, 4 algorithms were applied in the analysis: LR, DT, RF, and XGBoost. The importance of the variables age, BMI, and subjective recognition of body type as extracted from LR was 212.56, 173.26, and 138.01, respectively. Furthermore, the importance of the variables BMI and dietary condition as extracted from DT was 35.50, 7.07, and 5.53, respectively. The importance of the variables BMI, obesity, and age as extracted from RF was 7.07, 2.99, and 2.80, respectively. Finally, the importance of the variables status of drinking, weight control, and age as extracted from XGBoost was 6.34, 5.81, and 3.06, respectively (Table 2). To summarize, we found age, BMI, obesity, and the subjective recognition of body type to be the most important common variables.

**Table 2:** Analysis of the factors influencing metabolic syndrome using machine learning techniques.

Algorithm and variable name			Feature importance
**Logistic regression**			
	Age	212.56	
	BMI	173.26	
	Subjective body shape recognition	138.01	
	Amount of alcohol consumption at a time	87.98	
	Type of longest job	72.51	
	Subjective health status	71.79	
	Diagnosis of osteoarthritis	68.84	
	Diagnosis of arthritis	62.79	
	Daily activities	60.61	
	Status of other cancer treatments	57.59	
**Decision tree**			
	Age	30.50	
	BMI	7.07	
	Dietary conditions	5.53	
	Duration of walking	2.00	
	Age at which alcohol consumption began	1.99	
	Modified working hours	1.37	
	Status of smoking	1.31	
	Frequency of binge drinking	1.15	
	Frequency of eating out	0.89	
	Region	0.74	
**Random forest**			
	BMI	7.08	
	Obesity status	2.99	
	Age	2.80	
	Subjective body shape recognition	2.67	
	Type of longest job	1.14	
	Region	1.22	
	Age at which alcohol consumption began	1.11	
	Standard occupation classification	0.81	
	Education level	1.02	
	Walking days in a week	0.78	
**Extreme gradient boost**			
	Status of drinking	6.34	
	Weight control method	5.81	
	Driving under the influence during 1 y	3.06	
	Smoking cessation plan	1.91	
	Duration of disease state	1.64	
	Self-management	1.60	
	Age	1.60	
	Status of nutrition display impact	1.56	
	Daily activities	1.44	
	Status of pulmonary tuberculosis diagnosis	1.18	

### Determining the Number of Latent Class Layers

LCA was used to determine 4 indices of the model’s goodness of fit: Bayesian information criteria, sample size–adjusted Bayesian information criteria, Lo-Mendell-Rubin adjusted likelihood ratio test, and bootstrapped likelihood ratio test. We determined the number of class layers through a preferential check of each measured model’s goodness-of-fit index. In particular, we increased the number of layers, as illustrated in Table 3, and used several influencing factors to reveal the presence of metabolic syndrome in single-person households, finally deciding on 4 latent classes.

**Table 3:** Model fit indices for the latent class analysis model (N=1371).

Group number	Model fit indices				Classification of latent class, n (%)				
	BIC^a^	SSABIC^b^	BLRT^c^	LMR^d^	1	2	3	4	5
1	20,874.18	20,782.18	0.000	0.000	508 (37.05)	863 (62.95)	N/A^e^	N/A	N/A
2	2050.91	20,366.14	0.000	0.000	655 (47.78)	298 (21.74)	418 (30.49)	N/A	N/A
3	20,276.99	20,089.57	0.000	0.000	354 (25.82)	329 (24)	320 (23.34)	368 (26.8)	N/A
4	20,266.98	20,087.23	0.519	0.506	310 (22.61)	186 (13.57)	319 (23.27)	306 (22.32)	250 (18.23)

^a^BIC: Bayesian information criteria.

^b^BLRT: bootstrapped likelihood ratio test.

^c^LMR: Lo-Mendell-Rubin adjusted likelihood ratio test.

^d^SSABIC: sample size–adjusted Bayesian information criteria.

^e^N/A: not applicable.

### Names and Characteristics of the Latent Classes

It is important to select latent class classification variables to identify the factors affecting metabolic syndrome in single-person households through an in-depth consideration of prior research results [22,23].

Therefore, to diagnose metabolic syndrome, we selected sex, age, smoking, alcohol consumption, walking, obesity, hypertriglyceridemia, high blood pressure, high blood glucose, abdominal obesity, and hypo–high-density lipoproteinemia. On the basis of the characteristics and response patterns of subclass types classified through the LCA, we named these categorized classes as follows: group 1: intense physical activity in early adulthood, group 2: hypertension among middle-aged female respondents, group 3: smoking and drinking among middle-aged male respondents, and group 4: obesity and abdominal obesity among middle-aged male respondents. Tables 4 and 5 present the characteristics and names of each sublayer type according to each latent class.

From the 1371 participants, groups 1, 2, 3, and 4 had 320 (23.34%), 368 (26.84%), 329 (24%), and 354 (25.82%) participants, respectively. First, group 1 was compared with the other 3 groups, with 300 (93.8%) of the 320 participants indicating that age was the most important factor. Moreover, 289 (90.3%) respondents walked >3 times a week, which was substantially higher than that of the other groups. All the 5 diagnostic criteria for metabolic syndrome exhibited low rates, regardless of whether metabolic syndrome was present at 0%. In group 2, out of 368 respondents, 337 (91.6%) were female, and all participants in this group were in their middle adulthood. All the diagnostic criteria for metabolic syndrome exhibited low rates, whereas 47 (12.8%) participants had metabolic syndrome. In group 3, out of 329 respondents, 318 (96.7%) were male, which is more than the number of male respondents in other groups, and 250 (76%) respondents in this group were in their middle adulthood. The rate of smoking was high (n=249, 75.7%), and 181 (55%) participants reported a high frequency of alcohol consumption (>2 times/wk). In addition, 72 (21.9%) respondents had metabolic syndrome. In group 4, out of 354 participants, 255 (72%) participants were in middle adulthood. In terms of the diagnostic criteria for metabolic syndrome, 265 (74.9%) had hypertension, 306 (86.4%) were obese, 354 (100%) had abdominal obesity, and 232 (65.5%) had metabolic syndrome.

**Table 4:** Baseline characteristics according to latent class analysis–derived classes (N=1371).

Variable		Group 1 (n=320), n (%)	Group 2 (n=368), n (%)	Group 3 (n=329), n (%)	Group 4 (n=354), n (%)
**Sex**					
	Male	143 (44.7)	31 (8.4)	318 (96.7)	189 (53.4)
	Female	177 (55.3)	337 (91.6)	11 (3.3)	165 (46.6)
**Age (years)**					
	Early adulthood (19-39)	300 (93.8)	0 (0)	79 (24)	99 (28)
	Middle adulthood (40-64)	20 (6.2)	368 (100)	250 (76)	255 (72)
**Current smoking status**					
	Smoking	70 (21.9)	12 (3.3)	249 (75.7)	128 (36.2)
	Not smoking	250 (78.1)	356 (96.7)	80 (24.3)	226 (63.8)
**Frequency of drinking**					
	No drinking	18 (5.6)	132 (35.9)	34 (10.3)	76 (21.5)
	<4 times/mo	240 (75)	212 (57.6)	114 (34.7)	183 (51.7)
	>2 times/wk	62 (19.4)	24 (6.5)	181 (55)	95 (26.8)
**Frequency of walking days**					
	<3 times/wk	31 (9.7)	122 (33.2)	107 (32.5)	133 (37.6)
	>3 times/wk	289 (90.3)	246 (66.8)	222 (67.5)	221 (62.4)
**Obesity**					
	Low weight	32 (10)	15 (4.1)	11 (3.3)	0 (0)
	Normal	197 (61.6)	197 (53.5)	124 (37.7)	0 (0)
	Overweight	59 (18.4)	112 (30.4)	170 (51.7)	48 (13.6)
	Obese	32 (10)	44 (12)	24 (7.3)	306 (86.4)
**Dyslipidemia**					
	Yes	22 (6.9)	73 (19.8)	171 (52)	162 (45.8)
	No	298 (93.1)	295 (80.2)	158 (48)	192 (54.2)
**Hypertension**					
	Yes	17 (5.3)	238 (64.7)	164 (49.8)	205 (57.9)
	No	303 (94.7)	130 (35.3)	165 (50.2)	149 (42.1)
**Hyperlipidemia**					
	Yes	8 (2.5)	90 (24.5)	166 (50.5)	175 (49.4)
	No	312 (97.5)	278 (75.5)	163 (49.5)	179 (50.6)
**Abdominal obesity**					
	Yes	0 (0)	9 (2.4)	0 (0)	354 (100)
	No	320 (100)	359 (97.6)	329 (100)	0 (0)
**Hypo–high-density lipoproteinemia**					
	Yes	53 (16.6)	135 (36.7)	73 (22.2)	151 (42.7)
	No	267 (83.4)	233 (63.3)	256 (77.8)	203 (57.3)

**Table 5:** Latent classes of metabolic syndrome in South Korean single-person households.

Division	Group name	Participant (N=1371), n (%)	Diagnosis of metabolic syndrome, n (%)
Group 1	Intense physical activity in early adulthood	320 (23.3)	0 (0)^a^
Group 2	Hypertension among middle-aged female respondents	368 (26.8)	47 (12.8)^b^
Group 3	Smoking and drinking among middle-aged male respondents	329 (24)	72 (21.9)^c^
Group 4	Obesity and abdominal obesity among middle-aged respondents	354 (25.8)	232 (65.5)^d^

^a^n=320.

^b^n=368.

^c^n=329.

^d^n=354.

### Relationships Between Latent Class Groups and Metabolic Syndrome

We performed a binary LR to predict metabolic syndrome outbreaks in the categorized latent class groups (Table 6).

Regression analysis of the groups, as classified by the LCA (independent variables) and occurrence of metabolic syndrome (dependent variable) was significant (χ^2^_3_=521.7, *P*<.001). Further, the Cox and Snell coefficient of determination (*R*^2^=0.57), representing the model’s descriptive power, was 57%. The Hosmer-Lemeshow test results for the prediction model (*χ*²_3_=12.7, *P*=.49) demonstrated that no differences existed between the observed and predicted values.

The group with intense physical activity in middle adulthood was established as a reference category. In comparison, groups 2, 3, and 4 were 5.09 times (95% CI 3.15-14.91; *P*<.001), 8.99 times (95% CI 5.74-21.72; *P*<.001), and 17.67 times (95% CI 14.45-25.33; *P*<.001) more likely to experience metabolic syndrome, respectively.

**Table 6:** Relationship between latent groups and metabolic syndrome (N=1371)^a^.

Group	B (SE)	Odds ratio (95% CI)	*P* value
Intense physical activity in early adulthood	Reference^b^	Reference	Reference
Hypertension among middle-aged female respondents	2.08 (0.22)	5.09 (3.15-14.91)	.001
Smoking and drinking among middle-aged male respondents	2.94 (0.29)	8.99 (5.74-21.72)	.001
Obesity and abdominal obesity among middle-aged male respondents	3.75 (0.30)	17.67 (14.45-25.33)	.001

^a^*R*²=0.57, *χ*²_3_=12.7 in Hosmer-Lemeshow test; *P*=.49.

^b^Set as reference category in latent class analysis.

## Discussion

### Principal Findings

This study is the first to identify risk factors for metabolic syndrome in South Korean single-person households from multiple angles using LCA and machine learning techniques. The purpose of this study was to classify the risk factors for metabolic syndrome in single-person households using LCA and to identify the types and characteristics of the classified latent class. This paper describes metabolic health (BMI, body weight, body fat percentage, blood pressure, and blood sugar) among the physical and social characteristics of single-person households. There were more single-person households in middle adulthood (40-64 y) than in early adulthood (19-39 y). In this study, age, BMI, obesity, drinking, and body shape were found as potential risk factors for metabolic syndrome in single-person households. A cross-sectional study such as this is necessary because it can identify the factors that affect metabolic syndrome in single-person households in South Korea and determine which factors should be targeted through appropriate intervention [33].

Existing studies on metabolic syndrome were conducted mainly among older and middle-aged adults [34-37]. Among recent studies, several studies have confirmed the presence metabolic syndrome in the younger generation, suggesting that the metabolic syndrome morbidity rate among generations with various characteristics has increased [37,38]. On the basis of this, it was found that the diversity of single-person households could not be overlooked. Importantly, it has been reported that health habits have substantial influence on metabolic syndrome [39]. As health habits are already fixed in middle to late adulthood, it is difficult to expect changes in health behavior later; therefore, the prevention and management of metabolic syndrome in early adulthood should be considered [38-40]. Therefore, it is evident that modifying health habits is the most important step in treating or preventing metabolic syndrome.

In this study, to categorize the risk factors for metabolic syndrome in adult single-person households, the LR, DT, RF, and XGBoost algorithms, which are machine learning techniques, were applied to identify factors that affect the occurrence of metabolic syndrome in adult single-person households. In this analysis, variables such as age, BMI, obesity, alcohol consumption, and subjective body shape recognition were commonly derived. This suggests that the factors identified in previous studies as affecting metabolic syndrome in adult single-person households and the factors identified by applying machine learning techniques in this study are consistent with each other [30]. It is important to actively encourage physical activity to prevent metabolic syndrome [39]. In addition, it is necessary to develop a differentiated health management strategy using mobile health programs for single-person households in early adulthood with sustainable and compelling content relevant to their daily lives.

Unlike group 1, group 2 comprised mostly female respondents, primarily in the center of middle adulthood or older. In addition, this group had low rates of smoking and obesity and a high rate of hypertension. These results were consistent with those of previous studies, which indicated that high blood pressure in middle adulthood causes metabolic syndrome [40]. In addition, the rates of normal weight and overweight were the highest and second highest, respectively, in this group, which is consistent with the study by Kang et al [41], which reported that physical activity reduces hypertension and prevents metabolic syndrome among female individuals. This finding suggests that high blood pressure is an important risk factor for developing metabolic syndrome in single-person households [42].

Hypertension was an important risk factor, as seen in group 2. Thus, to prevent metabolic syndrome in group 2, it is important to develop and implement intervention programs for reducing blood pressure through diet and exercise therapy programs, encourage physical activity, and reduce obesity [43,44].

In group 3, the proportion of male respondents was significantly higher. In addition, the rate of smoking, frequency of alcohol consumption, and the rate of obesity were the highest in this group compared with the other groups. Moreover, sex and age were important risk factors for metabolic syndrome, which is consistent with the large proportion of middle-aged respondents in group 3. This group also exhibited characteristics of typical middle-aged workers, indicating the need to observe and manage smoking and alcohol consumption, especially among office workers [45]. These findings coincide with the finding of the study by Oh [46] that smoking facilitates metabolic syndrome, whereas its cessation prevents it among middle-aged male individuals. Thus, alcohol consumption and smoking were important risk factors for metabolic syndrome in group 3.

In this group, 21.9% (72/329) of the participants developed metabolic syndrome, and this group was 8.99 times more likely to develop metabolic syndrome than group 1. This corroborates the findings of Oh [46], as those in middle adulthood are more likely to be exposed to hypertension, hyperlipidemia, smoking, and alcohol consumption; hence, this group requires close monitoring and preventive nursing interventions. Moreover, although stress often leads to a desire to smoke and compels ex-smokers to begin smoking again, it is not fully clear as to why it is difficult to cease smoking [45,46]. Therefore, nursing interventions are needed to increase the motivation to quit smoking.

Further, another study discovered that the greater the stress, the higher the risk of health problems, such as smoking and depression [6,25]. Higher nicotine dependence demonstrates that smoking may be an inappropriate response if psychological problems such as stress and depression are not properly managed [12,44,45]. In addition, as Korean populations are often exposed to smoking when dining together and drinking socially, it is necessary to establish a culture of smoking cessation and changes in dining manners.

In group 4, the proportions of male respondents and female respondents were similar, with a high proportion of respondents in middle adulthood. Further, all respondents in the group exhibited obesity (based on the respondents’ BMI) or abdominal obesity (based on the respondents’ waist circumference). Obesity is also associated with the development of insulin resistance and beta-cell dysfunction, regardless of whether it is accompanied by abdominal obesity, which is consistent with prior literature [37,42]. Our results are also consistent with a report by Detournay et al [14], which revealed that obesity and abdominal obesity during female menopause may cause metabolic syndrome.

In group 4, metabolic syndrome was prevalent among 65.5% (232/354) of the respondents, and this group was 17.67 times more likely to develop metabolic syndrome than group 1. Moreover, the rates of hypertension, hyperglycemia, abdominal obesity, and hypo–high-density lipoproteinemia were higher than those in the other groups. As having at least 3 of the 5 criteria is an important basis for diagnosing metabolic syndrome, this is a critical factor [47].

This study’s LCA demonstrated that heterogeneous subgroups exist depending on metabolic syndrome risk factors, which is different from the results of most previous studies that focused on specific metabolic syndrome risk factors. We have proven that certain risk factors may have more prominent effects and affect certain age groups more strongly. Moreover, obesity and abdominal obesity were the most influential risk factors for metabolic syndrome in single-person households.

A national policy to promote physical activity is needed to prevent and manage metabolic syndrome in single-person households. In addition, strategies are needed to develop intervention programs for enhancing physical activity at any time or anywhere through mobile health and wearable devices; such programs would naturally integrate physical activity into daily life. Thus, it would be much more effective to develop and implement different risk-based intervention strategies for different individuals. It would be beneficial if customized mediations based on individual needs could be developed and implemented, taking into consideration subgroup characteristics instead of the collective metabolic syndrome risk factors. Therefore, rather than considering individuals with metabolic syndrome risk factors as a homogenous group and applying the developed interventions collectively, customized interventions should be developed considering the characteristics of each subgroup, and groups that share the same characteristics should be efficiently classified. Such interventions can be made much more effective if they incorporate strategies targeting each of the various risk factors for metabolic syndrome.

### Limitations

This study has several limitations. First, the NHANES questionnaire we used could not incorporate various variables. Due to annual changes in the survey questions, data were extracted that matched all 10 years of the survey questions. Second, as the object of investigation differed every year, tracking the longitudinal changes and progress of metabolic syndrome was a challenge. Third, although various machine learning techniques were used in this study, the most commonly used artificial neural network technique was not used. In the future, it will be necessary to conduct research applying deep learning methods such as artificial neural networks.

### Conclusions

This study is significant in that it is the first to use latent stratification analysis and machine learning techniques to identify the types and characteristics of potential subgroups classified based on potential metabolic syndrome risk factor indicators in adult single-person households. This study conducted a secondary analysis of data (2009-2018) from the NHANES hosted by the Korea Centers for Disease Control and Prevention, through which it classified and characterized risk factors for metabolic syndrome in adult single-person households.

In this study, machine learning techniques were applied to identify factors affecting metabolic syndrome in adult single-person households, which were identified as high parameters. In addition, the groups classified based on risk factors for metabolic syndrome in adult single-person households using LCA were intense physical activity in early adulthood, hypertension in middle-aged female respondents, smoking and drinking in middle-aged male respondents, and obesity and abdominal obesity in middle-aged male respondents. In addition, when confirming the difference between potential class groups according to the factors influencing metabolic syndrome, the 4 potential classes showed substantial differences in general characteristics such as education level, income level, frequency of dining out, dietary life, subjective health status, and subjective body shape recognition. In addition, when examining the prediction of the occurrence of metabolic syndrome for each group, it was found that the obesity and abdominal obesity in middle-aged male respondents group had the highest probability, indicating that it was the most susceptible high-risk group in terms of the occurrence of metabolic syndrome.

This study is meaningful as a new attempt to identify the factors influencing metabolic syndrome in adult single-person households by applying machine learning techniques, categorize risk factors for metabolic syndrome using LCA, and identify the characteristics of each latent class. Therefore, this study provides new knowledge and contributes to the prevention of metabolic syndrome in adult single-person households by identifying 4 latent classes through LCA and thus facilitating the development of customized interventions.

## Abbreviations

DT: decision tree
LCA: latent class analysis
LR: logistic regression
NHANES: National Health and Nutrition Examination Survey
RF: random forest
XGBoost: extreme gradient boost

## Data Availability

The data sets generated and analyzed during this study are available from the corresponding author upon reasonable request.
